# The hTERT Promoter Enhances the Antitumor Activity of an Oncolytic Adenovirus under a Hypoxic Microenvironment

**DOI:** 10.1371/journal.pone.0039292

**Published:** 2012-06-15

**Authors:** Yuuri Hashimoto, Hiroshi Tazawa, Fuminori Teraishi, Toru Kojima, Yuichi Watanabe, Futoshi Uno, Shuya Yano, Yasuo Urata, Shunsuke Kagawa, Toshiyoshi Fujiwara

**Affiliations:** 1 Department of Gastroenterological Surgery, Graduate School of Medicine, Dentistry and Pharmaceutical Sciences, Okayama University, Okayama, Japan; 2 Center for Gene and Cell Therapy, Okayama University Hospital, Okayama, Japan; 3 Oncolys BioPharma, Inc., Tokyo, Japan; University of Chicago, United States of America

## Abstract

Hypoxia is a microenvironmental factor that contributes to the invasion, progression and metastasis of tumor cells. Hypoxic tumor cells often show more resistance to conventional chemoradiotherapy than normoxic tumor cells, suggesting the requirement of novel antitumor therapies to efficiently eliminate the hypoxic tumor cells. We previously generated a tumor-specific replication-competent oncolytic adenovirus (OBP-301: Telomelysin), in which the human telomerase reverse transcriptase (*hTERT*) promoter drives viral E1 expression. Since the promoter activity of the *hTERT* gene has been shown to be upregulated by hypoxia, we hypothesized that, under hypoxic conditions, the antitumor effect of OBP-301 with the *hTERT* promoter would be more efficient than that of the wild-type adenovirus 5 (Ad5). In this study, we investigated the antitumor effects of OBP-301 and Ad5 against human cancer cells under a normoxic (20% oxygen) or a hypoxic (1% oxygen) condition. Hypoxic condition induced nuclear accumulation of the hypoxia-inducible factor-1α and upregulation of *hTERT* promoter activity in human cancer cells. The cytopathic activity of OBP-301 was significantly higher than that of Ad5 under hypoxic condition. Consistent with their cytopathic activity, the replication of OBP-301 was significantly higher than that of Ad5 under the hypoxic condition. OBP-301-mediated E1A was expressed within hypoxic areas of human xenograft tumors in mice. These results suggest that the cytopathic activity of OBP-301 against hypoxic tumor cells is mediated through hypoxia-mediated activation of the *hTERT* promoter. Regulation of oncolytic adenoviruses by the *hTERT* promoter is a promising antitumor strategy, not only for induction of tumor-specific oncolysis, but also for efficient elimination of hypoxic tumor cells.

## Introduction

Solid tumor tissues often contain hypoxic regions, in which the supply of oxygen and nutrition is reduced because of an immature vascular network, and in which there is rapid tumor progression [1]. Hypoxia is a critical microenvironmental factor that contributes to tumor angiogenesis, invasion, progression and metastasis [1], [2]. Indeed, hypoxic conditions have been shown to be associated with cancer progression and poor prognosis [3]–[5]. Furthermore, recent accumulated evidence suggests that hypoxia induces cancer progression-related characteristics such as epithelial-mesenchymal transition (EMT) [6], [7] and stemness properties [8]–[11] of tumor cells. Acquisition of such properties by tumor cells within hypoxic areas of tumor tissues would greatly contribute to tumor progression and recurrence.

Hypoxic tumor cells are known to be highly resistant to conventional chemoradiotherapy, leading to poor prognosis [5], [12]. To improve clinical outcome, novel antitumor agents that efficiently eradicate tumor cells under hypoxic conditions as well as under normoxic conditions are required. Oncolytic virotherapy has emerged as a promising novel antitumor therapy [13]. We previously generated a telomerase-specific replication-competent oncolytic adenovirus (OBP-301: Telomelysin), in which the human telomerase reverse transcriptase (*hTERT*) promoter element drives E1 gene expression. OBP-301 efficiently kills human cancer cells but not normal human somatic cells [14]. hTERT is a catalytic subunit of human telomerase and is highly expressed in tumor cells, but not in normal cells. hTERT expression closely correlates with telomerase activity [15]–[17]. Tumor-specific antitumor activity of OBP-301 against various types of human cancer cells with high telomerase activity has been demonstrated in both *in vitro* and *in vivo* settings [14], [18], [19]. Furthermore, the feasibility of OBP-301 for clinical use has been demonstrated in a recently completed phase I clinical trial in the USA of OBP-301 in patients with advanced solid tumors [20]. However, whether OBP-301 has an antitumor effect against hypoxic tumor cells remains unclear.

Hypoxia-inducible factor 1 (HIF-1) is a master transcription factor that is activated by hypoxia [1]. HIF-1 consists of α and β subunits and HIF-1α expression is tightly regulated by oxygen concentration. The HIF-1α protein is stabilized under hypoxic conditions, whereas it is immediately degraded under normoxic conditions. HIF-1α induces the expression of many down-stream target genes that are associated with cellular metabolism, proliferation, survival, apoptosis, neovascularization and migration [4]. The expression of many target genes is activated by HIF-1 through binding to a *cis*-acting hypoxia response element (HRE) located at their enhancer or promoter regions [4], [21], [22]. The *hTERT* gene is also a HIF-1-target gene. Two HREs that are present in the *hTERT* gene promoter are involved in hypoxia-mediated *hTERT* gene upregulation [23]–[25]. In contrast, it has also been shown that hypoxic conditions impair the replication of wild-type adenovirus in tumor cells [26], [27]. Based on these findings, we hypothesized that the cytopathic activity of OBP-301 that is regulated by the *hTERT* gene promoter would be much stronger against hypoxic tumor cells than that of wild-type adenovirus due to hypoxia-induced enhancement of OBP-301 virus replication.

In the present study, we evaluated whether hypoxic conditions affect the expression levels of hTERT and the coxsackie and adenovirus receptor (CAR) in human cancer cells. We next assessed the antitumor effects of OBP-301 and Ad5 against human cancer cells under normoxic or hypoxic conditions. We further evaluated the replication of OBP-301 within hypoxic areas of human xenograft tumors.

## Results

### Maintenance of human cancer cells under hypoxic conditions

**Figure 1:**
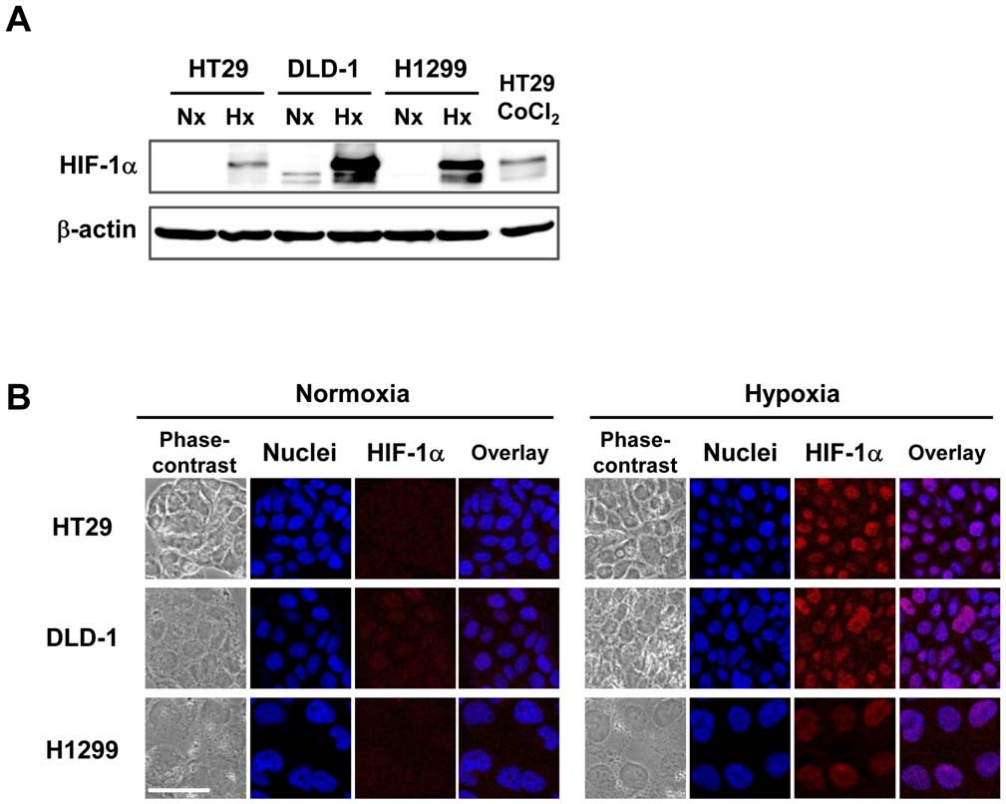
Increased HIF-1α expression in human cancer cells under hypoxic conditions. **A,** Western blot analysis of HIF-1α protein expression in human cancer cells (HT29, DLD-1 and H1299) under normoxic (Nx) or hypoxic (Hx) conditions. Cells were maintained under a normoxic (20% O_2_) or a hypoxic (1% O_2_) condition for 18 h. HT29 cells were also exposed to CoCl_2_ as a positive control. Cell lysates were subjected to Western blot analysis using an anti- HIF-1α antibody. β-actin was assayed as a loading control. **B,** Subcellular localization of HIF-1α expression in human cancer cells under normoxia or hypoxia was assessed using immunofluorescent staining. Cells cultured under a normoxic or a hypoxic condition for 18 h were stained with anti-HIF-1α antibody (red). Nuclei were counterstained with DAPI (blue). Scale bars = 50 µm.

A hypoxia chamber filled with a gas mixture of 1% O_2_, 5% CO_2_ and 94% N_2_ was used to maintain human cancer cells under hypoxic conditions. Human cancer cells were also maintained under normoxic conditions, consisting of 20% O_2_ and 5% CO_2_. To first confirm that the tumor cells were efficiently exposed to hypoxia in the chamber, the expression of HIF-1α, which is the main transcription factor induced by hypoxia [1], was evaluated using Western blot analysis. Consistent with HIF-1α induction by exposure to cobalt chloride (CoCl_2_), HIF-1α expression was strongly induced in human cancer cells (HT29, DLD-1, H1299) maintained in the hypoxia chamber (Fig. 1A). However, no, or slight, HIF-1α expression was detected under normoxic conditions. Moreover, using immunocytochemistry, we further confirmed that HIF-1α was expressed and accumulated in the nuclei of human cancer cells under hypoxic conditions, but not under normoxic conditions (Fig. 1B). These results indicate that human cancer cells are maintained under hypoxic conditions in the hypoxia chamber.

### Expression of hTERT and the adenovirus receptor in human cancer cells under hypoxic conditions

**Figure 2:**
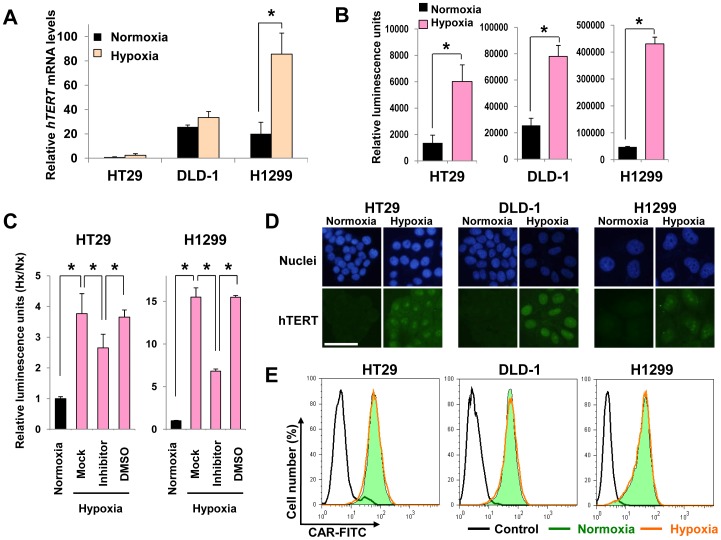
Effect of hypoxia on hTERT and CAR expression in human cancer cells. **A,** *hTERT* mRNA expression was assessed in human cancer cells that were maintained under normoxia (Nx) or hypoxia (Hx) for 18 h, using quantitative real-time RT- PCR analysis. The levels of *hTERT* mRNA were plotted as fold induction relative to the values of *hTERT* mRNA in HT29 cells incubated under normoxia, which was set at 1.0. Data are shown as mean values ± SD of triplicate experiments. Statistical significance (*) was determined as *P*<0.05 (Student's *t* test). **B** and **C**, *hTERT* gene promoter activity was assessed in human cancer cells that were transfected with the hTERT reporter vector (pGL3-hTERT) and then cultured under normoxia or hypoxia for 24 h, using luciferase reporter assay. The GFP expression vector (pCMV-EGFP) was used as a reporter for transfection efficiency, and the activities of hTERT promoter were determined as ratio of luciferase activity to GFP expression. Data are shown as mean values ± SD of triplicate experiments. Statistical significance (*) was determined as *P*<0.05 (Student's *t* test). **C**, HT29 and H1299 cells were treated with 30 µM HIF-1α inhibitor or DMSO solvent control in hypoxic condition. The levels of luminescence were plotted as fold induction relative to the values of luminescence in cancer cells incubated under normoxia, which were set at 1.0. **D**, Subcellular localization of hTERT protein expression in human cancer cells under normoxia or hypoxia was assessed using immunofluorescent staining. Cells cultured under a normoxic or hypoxic condition for 48 h were stained with anti-hTERT antibody (green). Nuclei were counterstained with DAPI (blue). Scale bars = 50 µm. **E,** Flow cytometric analysis of CAR expression in human cancer cells maintained under normoxia (green) or hypoxia (red) for 18 h. Cells were incubated with a mouse anti-CAR antibody followed by FITC-labeled rabbit anti-mouse IgG. An isotype-matched normal mouse IgG was used as a control (black).

OBP-301 contains the *hTERT* gene promoter, which allows tumor-specific regulation of the gene expression of *E1A* and *E1B* that are required for viral replication [14]. The activity of the *hTERT* gene promoter in human cancer cells has been shown to be upregulated under hypoxic conditions [23]–[25], suggesting that hypoxia would enhance OBP-301 replication through upregulation of *hTERT* gene promoter activity. To evaluate the effect of hypoxic conditions on the activity of the *hTERT* gene promoter in tumor cells, we first investigated the expression level of *hTERT* mRNA in human tumor cells under normoxic or hypoxic conditions by quantitative real-time RT-PCR analysis. The expression of *hTERT* mRNA was increased in all tumor cells under the hypoxic condition by 1.3 to 4.3-fold compared to the normoxic condition (Fig. 2A). Despite evident inductions of HIF-1α by hypoxia, the increases were not statistically significant in HT29 and DLD-1 cells. Because it is known that hTERT expression is regulated not only transcriptionally but also post-transcriptionally by alternative splicing [25], we further examined the effects of hypoxia on activity of exogenous *hTERT* gene promoter using luciferase reporter assay. Hypoxia activated the *hTERT* gene promoter by at least 3-fold compared to the *hTERT* gene promoter activity under normoxia (Fig. 2B). To further confirm hypoxia-induced *hTERT* promoter activation, we used chemical inhibitor of HIF-1α. The protein expression of HIF-1α and the activity of *hTERT* gene promoter were significantly decreased in HT29 and H1299 cells treated with 30 µM HIF-1α inhibitor LW6 and cultured in hypoxic condition (Fig. S1 and Fig. 2C). Moreover, we confirmed that hTERT protein was expressed and accumulated in nuclei of human cancer cells under hypoxic conditions by immunofluorescence staining (Fig. 2D). These results suggest that the *hTERT* gene promoter in OBP-301 is more strongly activated under the hypoxic condition than under the normoxic condition.

The infection efficiency of Ad5-based viral vectors depends mainly on the expression of the adenoviral receptor CAR in target cells [28]. Therefore, to evaluate whether hypoxic conditions affect the expression of CAR in tumor cells, we examined the expression level of CAR in all tumor cells under normoxic or hypoxic conditions by flow cytometry. CAR expression was clearly detected in all tumor cells tested: the percentage of CAR-positive cells was 99.5%, 99.3% and 98.8% for HT29, DLD-1 and H1299 cells, respectively (Fig. 2E). All tumor cell lines showed similar expression levels of CAR under normoxic and hypoxic conditions. These results indicate that tumor cells show high CAR expression under hypoxic conditions as well as under normoxic conditions.

### Antitumor activities of OBP-301 and Ad5 against hypoxic tumor cells

**Figure 3:**
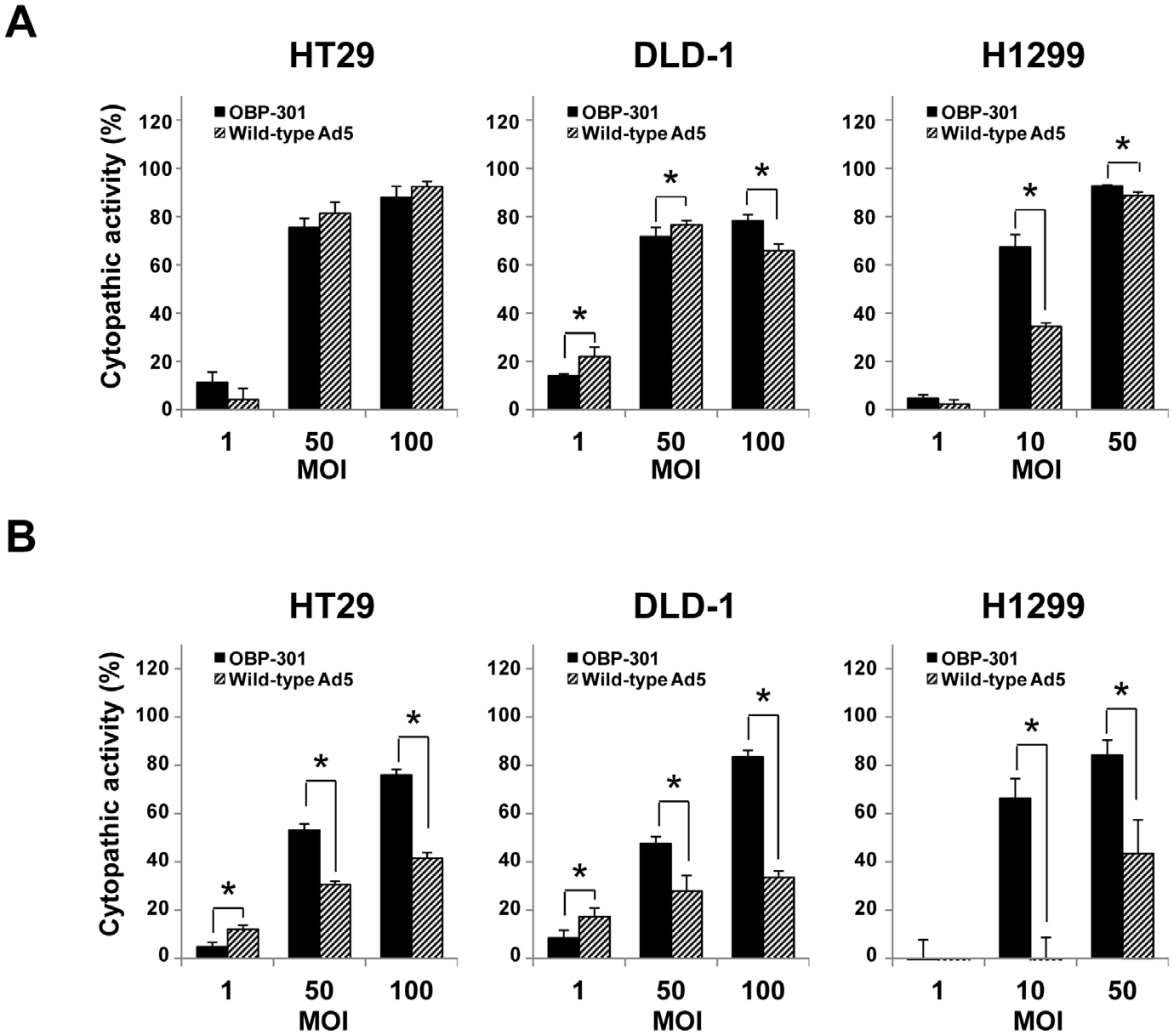
Cytopathic effect of OBP-301 and wild-type adenovirus serotype 5 (Ad5) under normoxic or hypoxic conditions. Cells were infected with OBP-301 (solid bars) or wild-type Ad5 (diagonal bars) at the indicated MOIs under normoxic (**A**) or hypoxic (**B**) conditions for 3 days. Cell viability was determined using an XTT assay. Cell viability was calculated relative to that of mock-treated cells, whose viability was set at 100%. Cytopathic activity was further calculated using the following formula; Cytopathic activity (%) = 100 (%) – cell viability (%). The results shown are the mean values ± SD of quadruplicate experiments. Statistical significance (*) was determined as *P*<0.05 (Student's *t* test).

To explore the potential antitumor activities of the telomerase-dependent oncolytic adenovirus OBP-301 against normoxic and hypoxic tumor cells, we investigated the cytopathic activities of OBP-301 and Ad5 against tumor cells under normoxic or hypoxic conditions. Under normoxic conditions, the cytopathic activities of OBP-301 and Ad5 against HT29 and DLD-1 cells were very similar. The cytopathic activity of OBP-301 against H1299 cells was significantly higher than that of Ad5 at a low dose of infection, whereas it was similar to that of Ad5 at a high dose of infection (Fig. 3A). In contrast, under hypoxic conditions, the cytopathic activity of OBP-301 against all tumor cells was significantly higher than that of Ad5, especially at a high dose of infection (Fig. 3B). To further evaluate the antitumor activities of OBP-301 and Ad5 against hypoxic tumor cells, the 50% inhibiting dose (ID_50_) values of OBP-301 and Ad5 under a hypoxic or a normoxic condition were calculated. The calculated ID_50_ values indicated that the cytopathic activity of OBP-301 against all tumor cells under hypoxic conditions was higher than that of Ad5, although the cytopathic activities of OBP-301 and Ad5 were very similar under normoxic conditions (Table 1). These results suggest that the cytopathic activity of OBP-301 against hypoxic tumor cells is more efficient than that of Ad5.

**Table 1:** Comparison of the ID_50_ values of OBP-301 and Ad5 against human cancer cells under normoxia and hypoxia.

Cell lines	Viruses	ID_50_ valuea) (MOI)		Ratiob) (Hx/Nx)
		Normoxia	Hypoxia	
HT29	OBP-301	20.2±1.5	51.7±7.3	2.5
	Ad5	25.6±4.3	212.8±52.0	9.4
DLD-1	OBP-301	8.2±2.7	26.3±10.3	3.0
	Ad5	6.6±1.9	2359.6±440.7	597.1
H1299	OBP-301	8.1±1.2	5.3±0.9	0.8
	Ad5	15.1±0.9	18.8±7.8	1.3

a) The ID_50_ values of OBP-301 and Ad5 were calculated from the data of the XTT assay at day 3 after infection. Data are shown as the mean values ± SE of triplicate experiments.

b) The ratio was calculated by division of the ID_50_ value under hypoxia (Hx) by the ID_50_ value under normoxia (Nx).

### Increased replication of OBP-301 compared to that of Ad5 under hypoxic conditions

**Figure 4:**
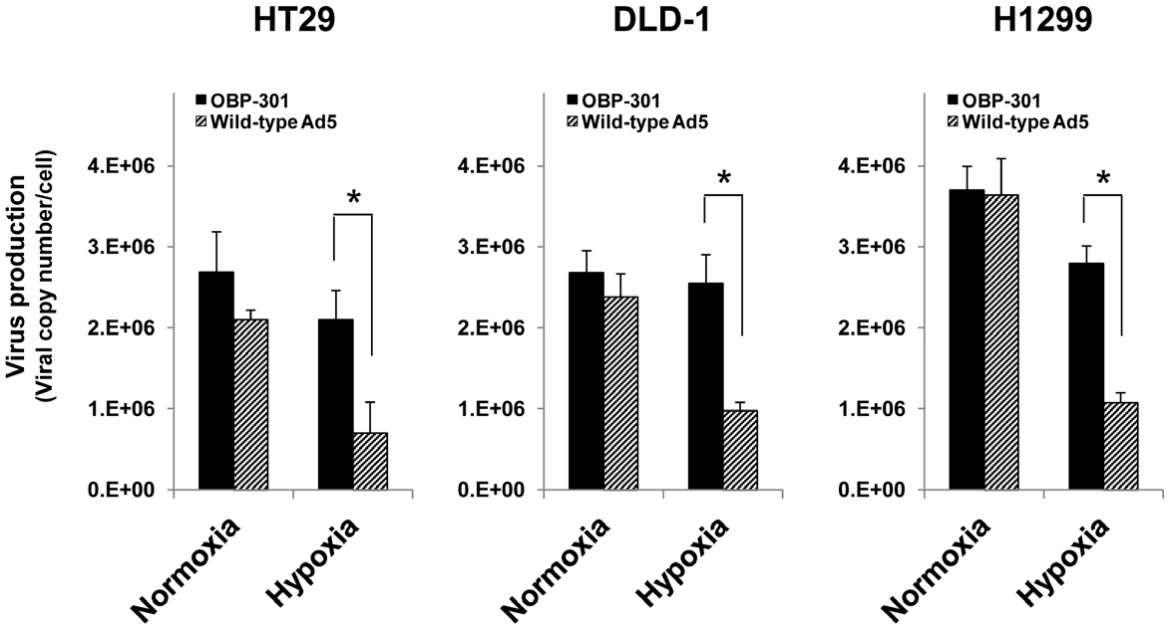
Quantification of viral DNA replication in human cancer cells under normoxia or hypoxia. The indicated human cancer cells were infected with OBP-301 or Ad5 at an MOI of 50 PFU/cell for 1 h, and were further incubated under normoxic (Nx) or hypoxic (Hx) conditions for 48 h. After incubation, cells were harvested and counted. *E1A* copy number in the cells at 48 h after incubation under normoxia or hypoxia was analyzed by quantitative PCR analysis. The amount of virus production was defined as the value of the *E1A* copy number relative to the number of cancer cells. Data are shown as the mean values ± SE of triplicate experiments. Statistical significance (*) was determined as *P*<0.05 (Student's *t* test).

We next examined the ability of OBP-301 and Ad5 to replicate in HT29, DLD-1 and H1299 cells, which showed almost similar sensitivity to OBP-301 and Ad5 under normoxic conditions but different sensitivity under hypoxic conditions (Fig. 3). The replication ability of OBP-301 and Ad5 was quantified by measuring viral E1A DNA in tumor cells infected with OBP-301 or Ad5 using quantitative real-time PCR analysis. Under normoxic conditions, the amount of virus production was very similar in tumor cells after infection with OBP-301 or Ad5 (Fig. 4). In contrast, under hypoxic conditions, viral production was significantly increased in OBP-301-infected tumor cells compared to Ad5-infected cells (Fig. 4). These results suggest that the replication of OBP-301 within hypoxic tumor cells is more efficient than that of Ad5.

### OBP-301-mediated E1A expression in the hypoxic regions of xenograft tumor tissues

**Figure 5:**
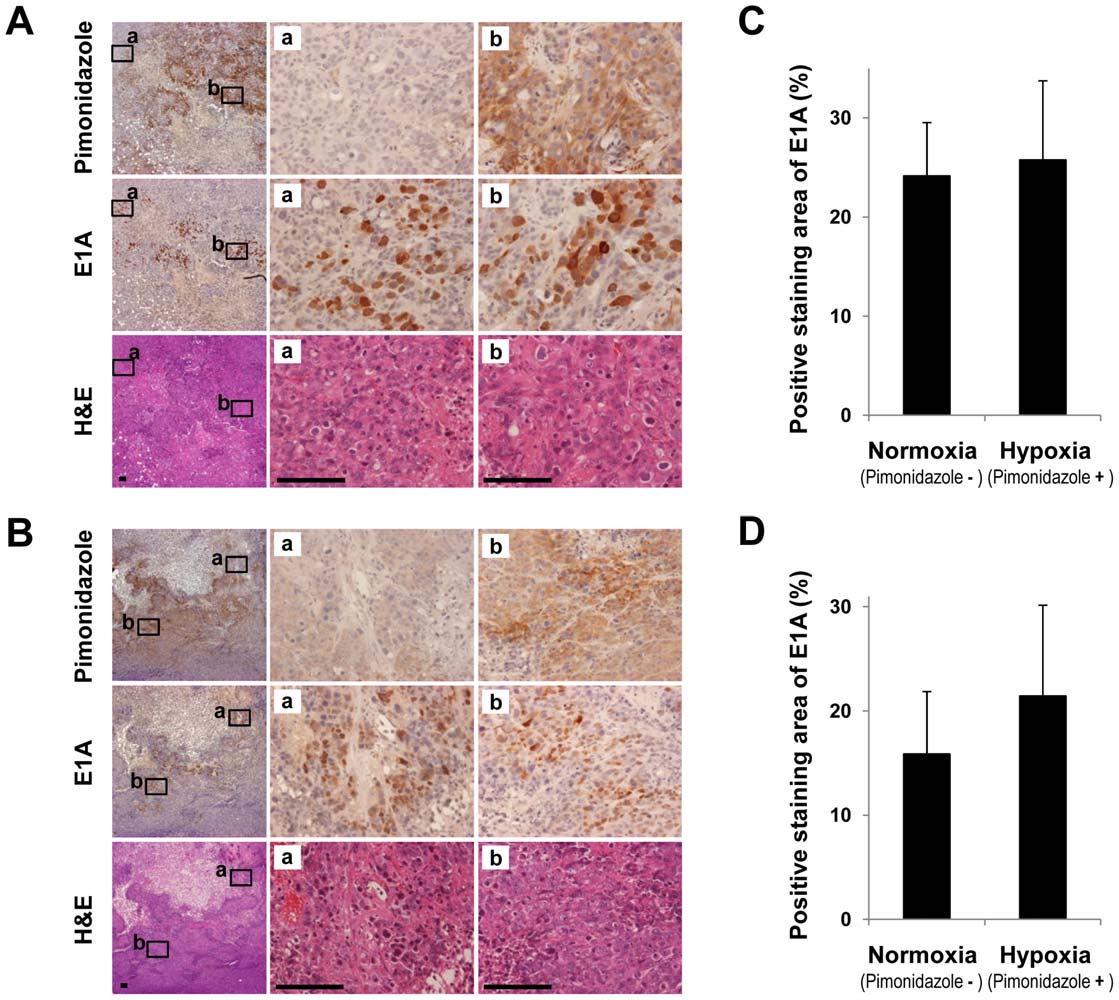
E1A expression in hypoxic areas of human xenograft tumors intratumorally injected with OBP-301. HT29 (**A** and **C**) and DLD-1 (**B** and **D**) tumor cells (5×10^6^cells/mouse) were injected subcutaneously into the flank of athymic nude mice. Two weeks after inoculation, OBP-301 (1×10^8^ PFU/tumor) was injected into the tumor for three cycles every 2 days. One day after final administration of OBP-301, the mice were intraperitoneally injected with the hypoxia marker pimonidazole hydrochloride (120 mg/kg). Thirty minutes after injection of pimonidazole hydrochloride, the mice were sacrificed and the tumors were harvested. Paraffin-embedded sections of HT29 and DLD-1 tumors were stained with hematoxylin and eosin (H&E). Tumor sections were also immunostained with an anti-pimonidazole antibody and an anti-adenovirus E1A antibody. **A** and **B**, Middle (**a**) and right (**b**) panels are higher magnifications of the boxed regions in the left panels. Original magnification: ×4 (left panels), ×40 (middle and right panels). Scale bars = 100 µm. **C** and **D**, Quantitative analysis of the E1A-positive areas in the normoxic and hypoxic regions of human xenografts tumor tissues. Data are shown as mean values ± SD of quadruplicate experiments.

To investigate whether OBP-301 actually replicates in the hypoxic regions of tumor tissues, we examined HT29 and DLD-1 xenograft tumors after intratumoral injection of OBP-301. OBP-301-mediated E1A protein expression was assessed in HT29 and DLD-1 xenograft tumors by immunohistochemistry. Hypoxic areas in tumor tissues were detected by immunohistochemical analysis of the exogenous hypoxic marker, pimonidazole hydrochloride. OBP-301-mediated E1A was expressed in the normoxic regions (Fig. 5Aa and 5Ba) and the regions that were confirmed to be hypoxic by detection of pimonidazole expression (Fig. 5Ab and 5Bb). Moreover, the quantitative image analysis of immunohistochemical stains showed that the E1A-positive areas were almost equal in pimonidazole-negative and pimonidazole-positive regions (Fig. 5C and 5D). These results suggest that OBP-301 replicates in hypoxic tumor cells within the hypoxic areas of tumor tissues.

## Discussion

Hypoxic microenvironments contribute to tumor invasion, progression, metastasis and resistance to conventional antitumor therapy, such as chemotherapy and radiotherapy, leading to poor prognosis [1]–[5]. The development of novel antitumor therapies that efficiently eliminate hypoxic tumor cells is an urgent issue for improvement of the clinical outcome of cancer patients. Although adenovirus-based oncolytic virotherapy has recently emerged as a promising antitumor therapy, a hypoxic microenvironment has been shown to reduce the replication of wild-type adenovirus in target tumor cells [26], [27]. Therefore, efficient replication of an oncolytic adenovirus under hypoxic conditions is a critical factor for the eradication of hypoxic tumor cells. In this study, our goal was to assess whether the telomerase-specific oncolytic adenovirus OBP-301 that is regulated by the *hTERT* gene promoter shows cytopathic activity against human tumor cells under hypoxic conditions. We demonstrated that the cytopathic activity of OBP-301 against hypoxic tumor cells was much stronger than that of wild-type adenovirus (Fig. 3 and Table 1). Hypoxia-mediated activation of the *hTERT* gene promoter was involved in the enhancement of virus replication in hypoxic tumor cells (Fig. 2 and 4). These results suggest that the *hTERT* gene promoter is useful for regulation of the replication of oncolytic adenoviruses in tumor cells in a hypoxic microenvironment.

The replication of OBP-301 depends on the activity of the *hTERT* gene promoter, which contains two HREs and is activated by HIF-1α under hypoxic conditions [23]–[25]. Hypoxic conditions that induced nuclear accumulation of HIF-1α (Fig. 1) upregulated *hTERT* gene promoter activity in human cancer cells (Fig. 2B and 2C). Consistent with this *hTERT* gene promoter activation, OBP-301 replication was significantly higher than that of Ad5 with the endogenous *E1* promoter (Fig. 4). These findings suggest that hypoxia enhances OBP-301 virus replication through HIF-1α-mediated activation of the *hTERT* gene promoter.

Recently, oncolytic virotherapy has garnered interest as potential therapeutic strategy for hypoxic tumors [29]. A hypoxia-responsive promoter that is upregulated by HIF-1 has been used for the tumor-specific replication of an oncolytic adenovirus [30]–[32]. Although an oncolytic adenovirus that is regulated by a hypoxia-responsive promoter will also be effective against hypoxic tumor cells following HIF-1 activation, non-hypoxic tumor cells in which HIF-1 is not activated may be less sensitive to these viruses. In contrast, the *hTERT* gene promoter-regulated oncolytic adenovirus OBP-301 would be effective against both hypoxic and normoxic tumor cells through hTERT activation.

The infection efficacy of Ad5-based oncolytic adenoviruses has been suggested to depend mainly on the expression level of CAR on the target cell surface [28]. Hypoxia has been shown to downregulate CAR expression in tumor cells in a HIF-1α dependent manner [33]. However, in the present study, high CAR expression was maintained in all of the human cancer cells tested, even under hypoxic conditions (Fig. 2E). These results are consistent with a previous report [27], which demonstrated that hypoxia has no influence on adenoviral infectivity of target cancer cells. The expression levels of integrin αvβ3 and αvβ5 are also involved in the infection efficacy of adenoviruses [34]. Previous reports have shown that hypoxia upregulates the expression levels of integrin αvβ3 and αvβ5 in tumor cells [35], [36]. These results suggest that hypoxic conditions would mainly suppress the replication of an adenovirus rather than the infection efficiency of the adenovirus.

Tumor tissues frequently contain hypoxic areas due to an immature vascular network. Various exogenous and endogenous hypoxia-related proteins have recently been developed as markers for identification of hypoxic regions of tumor tissues. Increased HIF-1 expression is a useful endogenous marker of hypoxic areas close to blood vessels. Expression of the exogenous hypoxia marker, Pimonidazole, is as effective a marker as HIF-1 for the detection of severely hypoxic regions [37]. In this study, OBP-301-mediated E1A expression was detected in pimonidazole-positive regions as well as normoxic regions (Fig. 5). These results indicate that the telomerase-specific oncolytic adenovirus OBP-301 could infect and replicate in tumor cells under a hypoxic microenvironment including in tumor cells in which HIF-1 was active.

Recent advances in our knowledge of tumor microenvironments have provided evidence that hypoxic tumor cells contribute to cancer progression. For example, hypoxia activates the metastatic potential of tumor cells by inducing EMT [6], [7] and facilitates the maintenance of cancer stem cells [8]–[11]. Therefore, the complete elimination of hypoxic tumor cells with metastatic and stemness properties is important for improvement of the clinical outcome of cancer patients. Recent reports have suggested that tumor cells undergoing EMT show reduced CAR expression [38], [39], suggesting that tumor cells undergoing EMT are less sensitive to oncolytic adenovirus infection. Further study to investigate the cytopathic effect of OBP-301 in tumor cells undergoing EMT is warranted. In contrast, recent reports have shown that an oncolytic adenovirus induces oncolytic cell death in cancer stem cells [40]–[43]. Cancer stem cells have recently been shown to have increased hTERT expression compared to non-cancer stem cells [44], [45]. Consistent with this high hTERT expression in cancer stem cells, Hemminki *et al*. has suggested that an oncolytic adenovirus that is regulated by specific promoters for hTERT, cyclooxygenase-2 or multidrug resistance, shows efficient cytopathic activity against human breast cancer stem cells [46]. Thus, the hTERT promoter-regulated oncolytic adenovirus OBP-301 may have the potential to eliminate highly progressive tumor cells in a hypoxic microenvironment, thereby contributing to the improvement of its therapeutic benefit against malignant tumors.

In conclusion, we have clearly demonstrated that the antitumor effect of the telomerase-specific oncolytic adenovirus OBP-301 against tumor cells in a hypoxic microenvironment is much stronger than that of a wild-type adenovirus. Regulation of virus replication by the *hTERT* gene promoter would be an effective antitumor strategy that would enhance the cytopathic activity of an oncolytic adenovirus against hypoxic tumor cells.

## Materials and Methods

### Cell lines

The human colorectal cancer (DLD-1 and HT29) and non-small cell lung cancer (H1299) cell lines were purchased from the American Type Culture Collection (ATCC) (Manassas, VA, USA). Although cell lines were not authenticated by the authors, cells were immediately expanded after receipt and stored in liquid N_2_. Cells were not cultured for more than 5 months following resuscitation. DLD-1 and H1299 cells were propagated as monolayer cultures in RPMI-1640 medium. HT29 was grown in McCoy's 5A medium. The transformed embryonic kidney cell line 293 obtained from the ATCC was maintained in Dulbecco's modified Eagle's medium containing high glucose (4.5 g/L). All media were supplemented with 10% heat-inactivated fetal calf serum, 100 units/ml penicillin G and 100 µg/ml streptomycin. To maintain human cancer cells under hypoxic conditions, the cells were incubated in a hypoxic chamber (Modular Incubator Chamber; Billups-Rothenberg, Del Mar, CA, USA) filled with a gas mixture of 1% O_2_, 5% CO_2_ and N_2_. The cells were also incubated under normoxic conditions at 37°C in a humidified atmosphere with 5% CO_2_ and 20% O_2_. HIF-1α inhibitor LW6 was purchased from Calbiochem (San Diego, CA, USA) and used at the concentration of 30 µM.

### Recombinant adenoviruses

The recombinant replication-selective, tumor-specific adenovirus OBP-301 (Telomelysin), in which elements within the *hTERT* gene promoter drive the expression of *E1A* and *E1B* genes linked with an internal ribosome entry site, was previously constructed and characterized [14]. The wild-type Ad5 was used as a control vector. OBP-301 and Ad5 were generated in 293 cells and purified by cesium chloride step-gradient ultracentrifugation. Their infectious titers were determined by a plaque-forming assay using 293 cells. The ratios of viral particle/plaque-forming unit of OBP-301 and Ad5 are 26 and 27, respectively. Viruses were stored at −80°C.

### Western blot analysis

Cells were maintained under a hypoxic or a normoxic condition for 18 h or 24 h. Whole cell lysates were then prepared in a lysis buffer (10 mM Tris (pH 7.5), 150 mM NaCl, 1 mM EDTA, 10% glycerol, 0.5% NP40) containing a protease inhibitor mixture (Complete Mini; Roche, Indianapolis, IN, USA). Lysates were electrophoresed on 4%–7% SDS polyacrylamide gels and proteins were transferred to polyvinylidene difluoride membranes (Hybond-P; GE Healthcare, Buckinghamshire, UK). The primary antibodies used for Western blotting were: mouse anti-HIF-1α monoclonal antibody (mAb) (BD Biosciences, San Diego, CA, USA) and mouse anti-β-actin mAb (Sigma, St. Louis, MO, USA). Horseradish peroxidase-conjugated antibody against mouse IgG (GE Healthcare) was used as the secondary antibody. Immunoreactive bands on the blots were visualized using enhanced chemiluminescence substrates (ECL Plus; GE Healthcare).

### Immunofluorescence staining

Cells grown in chamber slides were washed twice with ice-cold PBS, and then fixed with cold 4% paraformaldehyde in PBS for 15 min on ice. The cells were permeabilized by incubation with 0.2% Triton X-100 in PBS for 5 min on ice and then blocked with 3% bovine serum albumin in PBS for 30 min at room temperature. The slides were subsequently incubated with mouse anti-HIF-1α mAb (BD Biosciences) or mouse anti-hTERT mAb (KYOWA Medex, Tokyo, JP) for 1 h at room temperature. After two washes with PBS, the slides were incubated with Alexa Fluor 488- or Alexa Fluor 568-labeled goat anti-mouse IgG antibody (Invitrogen, Carlsbad, CA, USA) for 1 h. The slides were further stained with 10 mg/ml 4′,6-diamidino-2-phenylindole (DAPI), mounted using Fluorescence Mounting Medium (Dako, Glostrup, Denmark), and then photographed using a fluorescence microscope (IX71; Olympus, Tokyo, Japan).

### Quantitative real-time RT-PCR analysis

Total RNA was extracted from cancer cells maintained under hypoxic or normoxic conditions for 18 h using the RNA-Bee regent (Tel-test; Friendswood, TX, USA). The *hTERT* mRNA copy number was determined by quantitative real-time reverse transcription-polymerase chain reaction (RT-PCR) using a LightCycler instrument and a LightCycler Telo*TAGGG* hTERT Quantification Kit (Roche Diagnostics, Bagel, Switzerland). Data analysis was performed using LightCycler Software. The expression of *hTERT* mRNA was defined from the threshold cycle (Ct), and relative expression levels were calculated after normalization with reference to the expression of porphobilinogen deaminase (PBGD).

### Transfection and luciferase reporter assay

Cells were seeded on 6-well plates at a density of 4×10^5^ cells/well and incubated overnight. Each cell line was transfected with 3 µg of hTERT reporter plasmid (pGL3-hTERT) and 3 µg of GFP expression vector (pCMV-EGFP) as a reporter for transfection efficiency, using Lipofectamin LTX (Invitrogen) following the manufacturer's recommendations. Cells were then incubated under normoxic or hypoxic conditions. After 24 h incubation, luciferase activity was determined using a Bright-Glo reagent (Promega Corporation, Madison, WI, USA). Results presented are the ratios of luciferase activity to GFP fluorescent intensity and the means of three independent experiments.

### Flow cytometric analysis

The cells that were maintained under hypoxic or normoxic conditions for 18 h were labeled with a mouse anti-CAR mAb (Upstate Biotechnology, Lake Placid, NY, USA) for 30 min at 4°C. An isotype-matched normal mouse IgG1 (Serotec, Oxfordshire, UK) was used as a negative control. The cells were then incubated with a fluorescein isothiocyanate (FITC)-conjugated rabbit anti-mouse IgG second antibody (Zymed Laboratories, San Francisco, CA, USA) and were analyzed using flow cytometry (FACSCalibur; Becton Dickinson, Mountain View, CA, USA).

### Cell viability assay

Cells were seeded on 96-well plates at a density of 1×10^4^ cells/well 20 h before viral infection. All cell lines were infected with OBP-301 or wild-type Ad5 at multiplicity of infections (MOI) of 0, 1, 5, 10, 50 or using 100 plaque-forming units (PFU)/cell. The cells were then incubated under normoxic or hypoxic conditions for 3 days. Cell viability was determined using a Cell Proliferation Kit II (Roche Diagnostics) that was based on a sodium 3′-[1-(phenylaminocarbonyl)-3,4-tetrazolium]-bis (4-methoxy-6-nitro) benzene sulfonic acid hydrate (XTT) assay according to the manufacturer's protocol. The cytotoxic activity and the ID_50_ value of each virus was calculated using cell viability data. Each experiment was performed in quadruplicate during the same day and repeated at least three times.

### *In vitro* virus replication assay

Cells were seeded on 6-well plates at a density of 3×10^5^ cells/well 20 h before viral infection and were infected with OBP-301 or wild-type Ad5 at an MOI of 50 for 1 h. Following removal of the viral inocula, the cells were further maintained under hypoxic or normoxic conditions and were then harvested at 48 h after virus infection. After cell counting, DNA was purified using the QIAmp DNA mini Kit (Qiagen, Hilden, Germany) according to the manufacturer's protocol. E1A copy numbers were determined by quantitative real-time PCR using the StepOnePlus Real Time PCR System (Applied Biosystems, Carlsbad, CA, USA) and TaqMan Gene Expression Assays (Applied Biosystems). The sequences of the specific primers and probe used in this experiment were: E1A primers, 5′-CCT GAG ACG CCC GAC ATC-3′ and 5′-GGA CCG GAG TCA CAG CTA TCC-3′; E1A probe, 5′-FAM-CTG TGT CTA GAG AAT GC-MGB-3′. Data analysis was carried out using StepOne Software (Applied Biosystems).

### *In vivo* human xenograft tumor models

Animal experimental protocols were approved by the Ethics Review Committee for Animal Experimentation of Okayama University School of Medicine (Approval ID: OKU-2009051). The HT29 and DLD-1 cells (5×10^6^ cells per site) were inoculated subcutaneously into the flank of 5- to 6-week-old female BALB/c *nu*/*nu* mice (Japan SLC, Shizuoka, Japan). When the tumor size reached approximately 10 mm in diameter, OBP-301 was injected into the tumors at a dose of 1×10^8^ PFU/tumor every 2 days for three cycles. To detect hypoxic areas within tumor tissues, pimonidazole hydrochloride (Hypoxyprobe -1; Hypoxyprobe Inc., Burlington, MA, USA) was injected intraperitoneally at a dose of 120 mg/kg body weight 24 h after the final treatment. The mice were then sacrificed and the tumors were harvested 30 min after pimonidazole injection. Four mice were used for each group.

### Immunohistochemistry

Tumors were fixed in 10% neutralized formalin and embedded in paraffin blocks. Sections (4 µm) were prepared for hematoxylin/eosin staining and also for immunohistochemical examination. After deparaffinization and rehydration, antigen retrieval was performed by microwave irradiation in 10 mM citrate buffer (pH 6.0). After quenching of endogenous tissue peroxidase, tissue sections were incubated with mouse anti-adenovirus type 5 E1A mAb (BD Biosciences) and mouse anti-Hypoxyprobe-1 mAb (Hypoxyprobe Inc.). The sections were then incubated using the Histofine Mouse Stain Kit (Nichirei Biosciences, Tokyo, Japan). Immunoreactive signals were visualized by using 3,3′-diaminobenzidine tetrahydrochloride solution, and the nuclei were counterstained with hematoxylin. Signals were viewed under a microscope (BX50; Olympus). The percentage of the positive area in each field was analyzed using Image J software (version 1.45).

### Statistical analysis

Determination of significant differences among groups was assessed by using the Student's *t* test. *P*<0.05 was considered significant.

## Supporting Information

Figure S1 **Suppression of HIF-1α expression in human cancer cells under hypoxic conditions by HIF-1 inhibitor.** A, Western blot analysis of HIF-1α protein expression in human cancer cells (HT29 and H1299) under normoxic or hypoxic conditions. Cells were treated with 30 mM HIF-1α inhibitor or DMSO solvent control under hypoxic condition for 24 h. Cell lysates were subjected to Western blot analysis using an anti- HIF-1α antibody. β-actin was assayed as a loading control. **B,** Subcellular localization of HIF-1α expression in human cancer cells treated with 30 mM HIF-1α inhibitor or DMSO solvent control under hypoxia was assessed using immunofluorescent staining. Cells cultured under a hypoxic condition for 24 h were stained with anti-HIF-1α antibody (red). Nuclei were counterstained with DAPI (blue). Scale bars = 50 µm.
(TIF)
