# Supplementary figures and images for: The hTERT Promoter Enhances the Antitumor Activity of an Oncolytic Adenovirus under a Hypoxic Microenvironment
Source: PLoS One. 2012 Jun 15;7(6):e39292. doi: 10.1371/journal.pone.0039292 (PMC3376103; doi:10.1371/journal.pone.0039292)

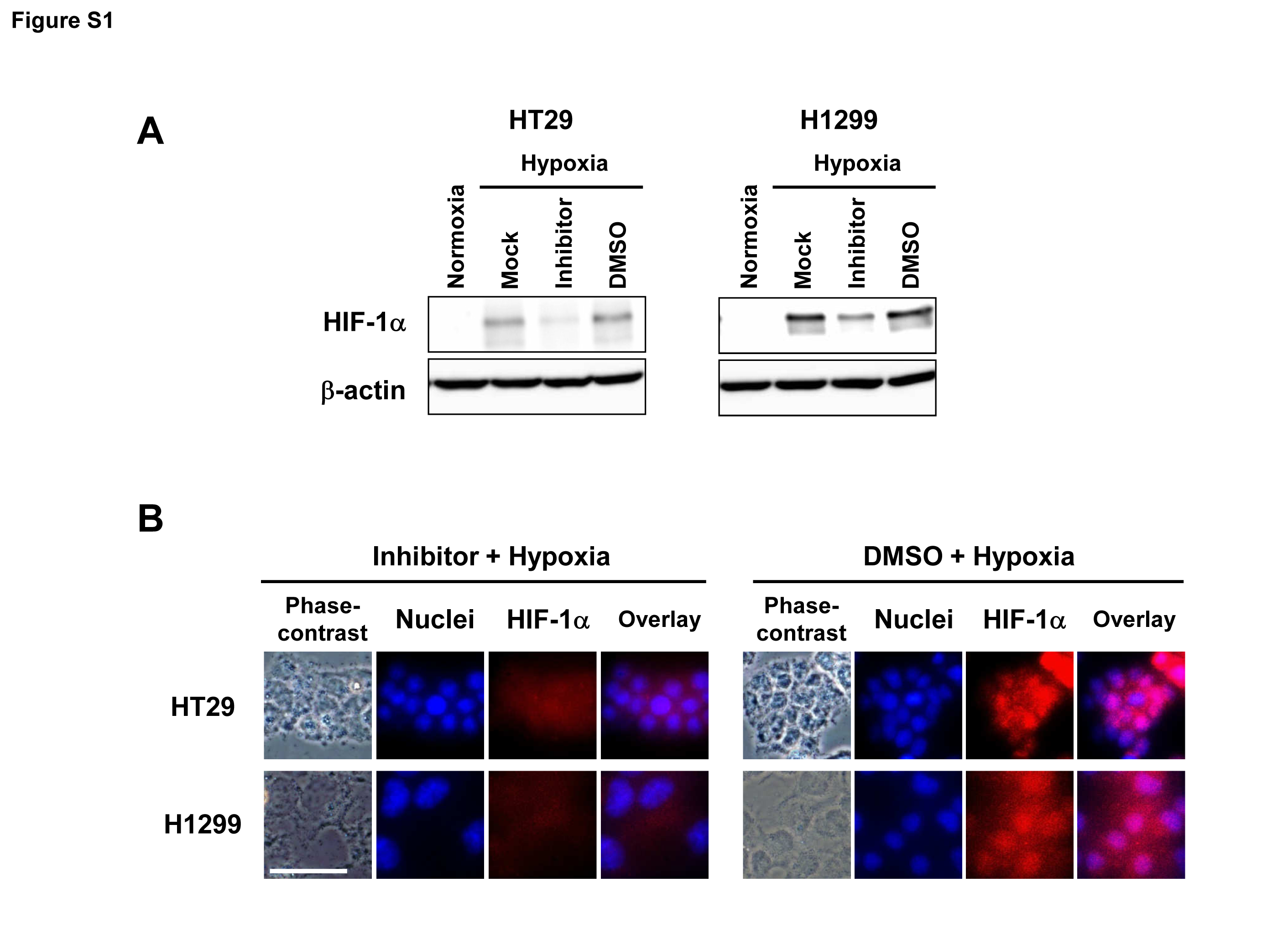

Supplement: Figure S1 — Suppression of HIF-1α expression in human cancer cells under hypoxic conditions by HIF-1 inhibitor. A, Western blot analysis of HIF-1α protein expression in human cancer cells (HT29 and H1299) under normoxic or hypoxic conditions. Cells were treated with 30 mM HIF-1α inhibitor or DMSO solvent control under hypoxic condition for 24 h. Cell lysates were subjected to Western blot analysis using an anti- HIF-1α antibody. β-actin was assayed as a loading control. B, Subcellular localization of HIF-1α expression in human cancer cells treated with 30 mM HIF-1α inhibitor or DMSO solvent control under hypoxia was assessed using immunofluorescent staining. Cells cultured under a hypoxic condition for 24 h were stained with anti-HIF-1α antibody (red). Nuclei were counterstained with DAPI (blue). Scale bars = 50 µm. (TIF) [file pone.0039292.s001.tif]
